# Evaluation of the Pharmacokinetics and Hepatoprotective Effects of Phillygenin in Mouse

**DOI:** 10.1155/2018/7964318

**Published:** 2018-08-23

**Authors:** Wei Song, Junjun Wu, Longjiang Yu, Zhihong Peng

**Affiliations:** ^1^Hubei Province Key Laboratory of Biotechnology of Chinese Traditional Medicine, Department of Life Science, National & Local Joint Engineering Research Center of High-Throughput Drug Screening Technology, Hubei University, Wuhan 430062, China; ^2^Institute of Resource Biology and Biotechnology, Department of biotechnology, College of Life Science and Technology, Huazhong University of Science and Technology, Wuhan 430074, China; ^3^Lab of Structure Biology and Medicinal Chemistry, Hubei University of Arts and Science, Xiangyang 441053, China

## Abstract

Phillygenin* is *a bioactive intergradient in* Osmanthus fragrans,* a well-known food additive and Chinese traditional medicine. This study was to investigate the hepatoprotective effects and pharmacokinetics of phillygenin. The hepatoprotective effect of phillygenin was assessed in carbon tetrachloride- (CCl_4_-) intoxicated mice by monitoring levels of serum and tissue biomarkers. The pharmacokinetics of phillygenin was evaluated in the mouse after oral (*po*, 24 mg / kg) or intravenous (*iv*, 12 mg/kg) administration. Results showed that phillygenin has a great hepatoprotective effect on CCl4-induced liver injury in mice owing to its antioxidant activity and inhibition on cytochrome P450 2E1(CYP2E1). After oral administration, phillygenin was efficiently absorbed with the oral bioavailability of 56.4%. Two metabolites, hydroxylated and dimethylated phillygenin, were identified in mouse urine. These results suggested that phillygenin could be explored as new and potential natural antioxidants and hepatoprotective agents.

## 1. Introduction

*Osmanthus fragrans* is a plant belonging to the* Oleaceae* family [1], which has been cultivated and distributed in East Asia, spanning a large geographic region through China to Japan [2].* Osmanthus fragrans* flower is not only valued as an additive for tea and various foods but also used as cosmetics for hair and skin and aromatic therapy [1]. In addition,* Osmanthus fragrans* flower has a wide range of pharmacological properties including antioxidation [3], neuroprotection [4], and antimicrobial effects [5]. Our lab has found that* Osmanthus fragrans* flower extract (OFE) showed a great hepatoprotective effect on CCl_4_-induced hepatic injury mice [2].

Phillygenin, extracted from* Osmanthus fragrans* flower for the first time by our group [6], has many medicinal properties. It can reduce blood lipid levels [7] and low density lipoprotein [8]. It is also useful in therapeutic and preventive applications in treating ONOO-related diseases [9]. Although phillygenin has many pharmacological activities, there is limited hepatoprotective information on phillygenin. What is more, to reflect the absorption and the first pass effect with which a drug is absorbed after oral administration, its oral bioavailability should be assessed, but the information of phillygenin pharmacokinetics is still limited [10, 11]. Present study primarily aims to evaluate the hepatoprotective of the OFE and its main bioactive component phillygenin on CCl4-induced hepatic injury mice. The pharmacokinetics and metabolism of phillygenin in mice are also studied.

## 2. Materials and Methods

### 2.1. Chemicals and Reagents

Phillygenin (purity > 98.6%) was extracted and purified from* Osmanthus fragrans* by our laboratory [6]. Ethylenediamine tetracetic acid (EDTA), pyrogallic acid, ammonium thiocyanate, potassium persulfate, ferrous chloride, ascorbic acid (purity > 98.0%), 2,4,6-tripyridyl-s-triazine and ferric chloride, diphenyl-2-picrylhydrazyl (DPPH), diethyldithiocarbamate, chlorzoxazone, 6-hydroxy chlorzoxazone, and *β*-Nicotinamide adenine dinucleotide 2′-phosphate reduced tetrasodium salt hydrate (NADPH) were purchased from Sigma-Aldrich (St. Louis, USA). All other chemicals and solvents were of chromatography grade. Human recombinant human CYP2E1 was purchased from BD Biosciences (San Diego, USA).

Liquid chromatography grade solvents were purchased from Sigma-Aldrich (St. Louis, USA). Purified water (Milli-Q Advantage, Millipore, Worcester, USA) was used for all preparations. The stock solution of phillygenin was prepared in acetonitrile and stored at −20°C. Bifendate (purity > 98.0%) (batch number: 110405, Zhejiang Medicine Co. Ltd., Xinchang Pharmaceutical Factory, Xinchang, China) was used as positive control in the hepatoprotective study.

### 2.2. Preparation of the OFE

The flower of* Osmanthus fragrans* was collected from Wuhan, Hubei Province, China, in October 2010. The fresh flowers were dried naturally and extracted by petroleum ether (*w:w*, 1:3) at room temperature for 2 hours using a shaker for three times. The extracts were filtrated and then dried by vacuum rotary evaporator to yield OFE. The OFE was dissolved in olive oil for the hepatoprotective study on animals.

### 2.3. Antioxidants Assay

#### 2.3.1. DPPH Radical-Scavenging Activity Assay

DPPH radical-scavenging activity was determined using a modified method [12, 13]. The reaction system contained 1.0 ml of DPPH solution (0.3 mM in methanol) and 2.5 ml sample solution (dissolved in methanol). The absorbance was detected at 515 nm after the mixture was shaken vigorously and incubated at room temperature for 30 min. The scavenging effect was determined as follows:

(1) $$\begin{array}{c} \text{DPPH\% inhibition}=\left[\;\frac{\left(A1-A2\right)}{A1}\;\right]\times 100, \end{array}$$

where A1 is the absorbance in the absence of the sample and A2 represents the absorbance in the presence of the sample.

#### 2.3.2. ABTS Radical-Scavenging Activity Assay

The procedures for ABTS assay followed the method [13, 14] with some modifications. 7.0 mM ABTS solution and 5.0 mM potassium persulfate solution were mixed in equal quantities and stored at room temperature in the dark for 14 h to get ABTS^+^ solution. The solution was then diluted by mixing 1 mL ABTS^+^ solution with 60 mL methanol to obtain an absorbance of 0.70 ± 0.02 units at 734 nm using the spectrophotometer. Phillygenin (200 *μ*L) was mixed with 3.0 mL of the ABTS^+^ solution for 15 min in a dark condition. The absorbance was detected at 734 nm on a biotech spectrophotometer (VT, US). The result was expressed as IC50 value.

### 2.4. Assessment of the Effect of Phillygenin on CYP2E1

To assess the potential of phillygenin to inhibit CYP2E1, the formation of chlorzoxazone 6-hydroxylation was used to assay the activity of CYP2E1 in the presence and absence of phillygenin. Seven phillygenin concentrations (0.01–10 *μ*M) were used. In brief, to determine the IC50 values, a mixture (200 *μ*L) containing phosphate buffer (50 mM, pH 7.4), chlorzoxazone (10 *μ*M), and CYP2E1 (40 pmol of protein/mL) was prepared for each. After preincubation at 37°C for 5 min, 10 *μ*L of NADPH (20 mM) was added to initiate the reaction. Positive control experiments were run in parallel by incubating each probe substrate at 37°C in duplicate with CYP2E1 in the absence (control) and the presence of diethyldithiocarbamate (a specific inhibitor of CYP2E1) [15]. The organic solvent was removed by drying of the solution in a speed vacuum concentrator (Genevac Ltd., Suffolk, UK). After incubation, 400 *μ*L acetonitrile containing internal standard was added to stop the reaction. The experimental samples were centrifuged at 10,000** ×** g for 20 min, and then the supernatants were transferred to new vials and concentrated by drying of the solution in a speed vacuum concentrator. The residues were redissolved in 100 *μ*L acetonitrile/water (50/50, v/v) and analyzed by ultra-performance liquid chromatography (UPLC) with multiple-reaction monitoring (MRM) detection.

Waters Acquity UPLC with a reversed column (Aquity UPLC C18, 1.7 *μ*m, 2.1mm i.d. × 100 mm) was used to analyze the samples. The flow rate was 0.5 mL/min with 90% A/10% B for 2 min, followed by a 7-min linear gradient to 10% A/90% B, then hold for 4 min (A = water, B = acetonitrile). Mass spectrometric experiments were carried out with a Waters TQD tandem quadrupole detector (Milford, MA, USA) using electrospray negative ionization mode. The capillary voltage, cone voltage, extractor voltage, and RF lens voltage were set at 2.8 kV, 35 V, 3.0 V, and 0.3 V, respectively. Nitrogen was used as desolvation gas (650 L/h) and cone gas (50 L/h). The source temperature was 150°C and desolvation temperature was 350°C. The MRM transition for chlorzoxazone 6-hydroxylation is 184→64.

IC50 values were determined by nonlinear regression with GraphPad Prism 5 software (GraphPadSoftware Inc., San Diego, CA). The* K*i values were determined by the following equation for competitive inhibition.

(2) $$\begin{array}{c} v=\frac{V\max\left[S\right]}{\left[S\right]+Km\left(1+\left[I\right]/Ki\right)} \end{array}$$

### 2.5. Pharmacodynamics Assays

#### 2.5.1. Animals

Mice (Male Kunming, 6-8 weeks old, 17-20 g body weight) were obtained from Hubei Experimental Animal Research Center, China. All animal experimental protocols involving animals were reviewed and approved by the institutional animal experimentation committee of Huazhong University of Science and Technology (No. 00019655, 01/11/2011).

#### 2.5.2. Hepatoprotective Effect of Osmanthus Fragrans Flower Extract and Phillygenin

Mice (n = 9 for each group) were separated into nine groups: normal control, CCl_4_ group, positive control, OFE (150, 300, 600 mg/kg), phillygenin (6, 12, 24 mg / kg). The normal control group received sterile distilled water. The positive control group mice were given bifendate by oral administration at the dose of 150 mg / kg once daily for seven consecutive days and induced by a single injection of CCl_4_ (ip, 10 mL / kg of 0.1% in olive oil) once a day from day 5 for three consecutive days. The phillygenin/OFE group mice were pretreated once a day with phillygenin by oral administration for seven consecutive days and also induced by CCl_4_ at the same time as the positive control group. The CCl_4_ group received sterile distilled water with normal mice chow and was induced by CCl_4_ from day 5 for three consecutive days. There are two mice dead occasionally for the CCl_4_ group, one mouse dead for 150 mg / kg OFE group and two mice for 6 mg / kg phillygenin group. All animals were sacrificed under mild ether anesthesia 16 h after last CCl_4_ injection, and blood samples were collected immediately.

Collected blood samples were placed at room temperature for 1 h and then centrifuged at 1000 × g for 10 min to obtain serum. Aspartate aminotransferase (AST) and alanine aminotransferase (ALT) levels were determined with commercial kits (Nanjing Jiancheng Biological Technology, Inc., China).

Liver samples were homogenized in Tris-HCl buffer (5 mM containing 2 mM EDTA, pH 7.4) to give 10% (w/v) liver homogenates. The homogenates were then centrifuged at 1000 × g for 10 min at 4°C, and the supernatants were used immediately for the determination of antioxidant status. Activities of antioxidant defense enzymes, superoxide dismutase (SOD), malondialdehyde (MDA), and protein content of the homogenates were determined with commercial kits (Nanjing Jiancheng Biological Technology, Inc., China). Liver tissues for the histopathological analysis were fixed in 10% buffered formalin saline, processed by routine histology procedures, and embedded in paraffin. Tissue sections (4–5 *μ*m) were stained with hematoxylin and eosin, observed under the microscope (NIKON TS100, Japan), and recorded.

### 2.6. Pharmacokinetics Assays

#### 2.6.1. Samples Preparation

A 100-*μ*L aliquot of plasma was mixed with 2.5 *μ*L of internal standard (IS) in acetonitrile to a final concentration of 5 *μ*g / mL. The sample was centrifuged at 20 000 × g for 15 min. The supernatants from plasma samples were collected for UPLC-MRM analysis.

#### 2.6.2. UPLC-MS/MS Conditions

Phillygenin analysis was carried out on a Waters Acquity UPLC System (Waters Corporation, Milford, MA, USA) equipped with a binary solvent manager, an autosampler, a column heater, and a PDA e*λ* detector, scanning from 190 to 400 nm. Separation was carried out using an Acquity BEH Shield RP C18 column (1.7 *μ*m, 2.1 mm i.d. × 100 mm, Waters) eluted at 0.4 mL/min with 0-2 min at 90% A/10% B, then 2-12 min linear gradient from 90% A/10% B to 10% A/90% B, and 12 min to 15 min from 10% A/90% B to 10% A/90% B (A = water; B = acetonitrile).

Mass spectrometric experiments were performed on a Waters TQD tandem quadrupole detector (Milford, MA, USA) with MassLynx MS software. Phillygenin and the metabolites were analyzed in the negative ESI mode and data-dependence MS/MS scanning from m/z 100 to 1000. Conditions were as follows: capillary voltage 3.2 kV, cone voltage 25 V, extractor voltage 3 V, RF lens voltage 0.1 V, desolvation nitrogen gas flow rate 650 L/hr, cone nitrogen gas flow rate 50 L/h, source temperature 147°C, and desolvation temperature 350°C.

#### 2.6.3. Method Validation

(*1) Calibration, Precision, Accuracy, and Matrix Effect.* The calibration curve was prepared with 0.01-10 *μ*g/mL calibration standards in mouse plasma to determine the linear range. A calibration curve was also prepared in aqueous solution and compared to the plasma calibration curve to assess potential matrix effects. Quality control (QC) samples were prepared daily for three days at three levels (low, medium, and high) in mouse plasma. The preparation of QC samples was the same as described in samples preparation. Blank plasma was spiked with standard phillygenin at final concentrations corresponding to low QC of 0.01 *μ*g/mL, medium QC of 1 *μ*g/mL, and high QC of 10 *μ*g/mL in plasma.

(*2) Stability and Recovery.* To evaluate the stability of phillygenin in mouse plasma, low, medium, and high QCs were stored at −80°C for one month for long-term stability. Short-term room temperature (RT) stability of phillygenin in mouse plasma was assessed by analyzing QC samples stored at room temperature for 6 h. For freeze-thaw stability of phillygenin, each set of low, medium, and high QCs was prepared in triplicate and determined after three freeze-thaw cycles (−80°C to RT). The extraction efficiency of phillygenin was determined by dividing the analyte plasma concentration with the calculated aqueous QC concentration.

#### 2.6.4. Pharmacokinetics of Phillygenin in Mice

Mice (n = 6 per time point) were given a single oral dose of phillygenin at 24 mg / kg. Terminal blood samples were collected in heparin through the posterior vena cava at 0.5, 1, 2, 3, 4, 6, 9, 24, and 30 h. After iv injection of phillygenin at 12 mg / kg, blood samples were collected at 2, 5, 10, 20, and 40 min and 1, 2, 3, 4, 6, 18, and 24 h after administration. Blood samples were stored on ice and centrifuged at 1200 × g, 4°C for 10 min to collect plasma and then stored at −80°C until analysis.

#### 2.6.5. Pharmacokinetic Parameters and Statistical Analysis

The area under the mean concentration-time curve up to the last quantifiable sampling time (*AUC*_0-last_), volume distribution (Vd), clearance (Cl), initial concentration (C_0_), elimination half-life (E Half-life), absorption half-life (A Half-life), and distribution half-life (D Half-life) was calculated by the trapezoidal rule. *AUC*_0-*∞*_ was calculated as *AUC*_0-last +_ (*C*_last_/*k*), where *C*_last_ is the concentration at the last quantifiable sampling time and *k* is the elimination rate constant. Half-lives (*t*_(1/2)*α*_ and *t*_(1/2)*β*_) were determined from the linear segments of the initial or terminal linear portion of the concentration-time data by linear regression, where the slope of the line is the rate constant *k* and *t*_(1/2)*α*_ = ln⁡2/*k*. The oral bioavailability was calculated by the following equation:

(3) $$\begin{array}{c} \text{Oral bioavailability }\left(\;\%\;\right)=\frac{\left({AUC}_{po}\times dose_{iv}\right)}{\left({AUC}_{iv}\times dose_{po}\right)}\times 100\% \end{array}$$

#### 2.6.6. Metabolism of Phillygenin in Mouse Urine

Mice were housed in metabolic cages for the collection of urine. Mice fasted for 24 h but with access to water and then were given a single dose of phillygenin (24 mg/kg weight) by oral gavage. Mice urine were collected after administration of phillygenin for 24 h and centrifuged at 1,000× g for 10 min; the supernatants were collected and stored at −80°C until analysis.

## 3. Results

### 3.1. Antioxidant Activity of Phillygenin In Vitro

**Figure 1:**
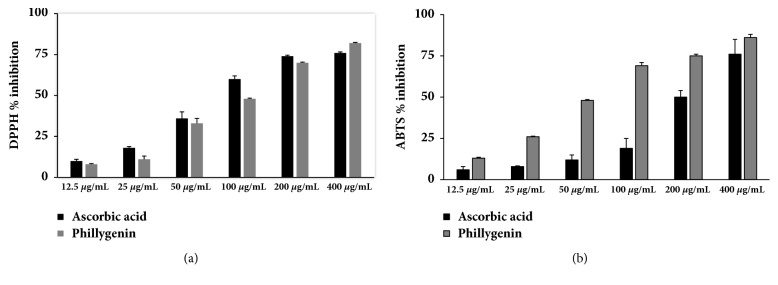
Evaluation of antioxidant activities of phillygenin using (a) DPPH and (b) ABTS methods. (n = 3).

Antioxidant activity of phillygenin was evaluated* in vitro*.  Figure 1 shows DPPH and ABTS radical-scavenging activities of phillygenin and ascorbic acid at different concentrations. The results demonstrated that both of phillygenin and ascorbic acid could eliminate DPPH and ABTS radicals in a dose-dependent manner. The quality of antioxidants was determined by IC50 values (antioxidant concentration that reduces the DPPH/ ABTS radical by 50%). The results show that the phillygenin had potent DPPH (IC50 102 ± 4.17 *μ*g/mL) and ABTS (IC50 49.0 ± 4.25 *μ*g/mL) free-radical scavenging activity.

### 3.2. Inhibition of CYP2E1 by Phillygenin

**Figure 2:**
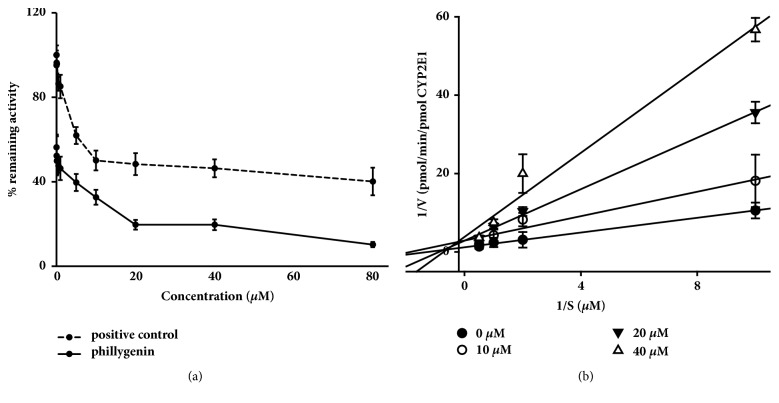
Inhibition of recombinant human CYP2E1 enzymes by phillygenin. (a) Chlorzoxazone at a concentration near its Km was incubated with CYP2E1 enzymes and cofactors in the presence of diethyldithiocarbamate (positive control) (0 to 80 *μ*M) or phillygenin (0 to 80 *μ*M). Each point represents the average of duplicate incubations. (b). Lineweaver-Burk plots showing the inhibition of CYP2E1-catalyzed chlorzoxazone 6-hydroxylation by phillygenin (0, 10, 20, and 40 *μ*M) in human recombinant human CYP2E1 enzymes. Each data point represents the average of duplicate experiments.

Inhibition studies allowed the calculation of IC_50_s and *K*_i_ values and the determination of the type of inhibition for CYP2E1. The results indicated that phillygenin showed potent inhibition of CYP2E1 with IC_50_ of 4.50 *μ*M (Figure 2(a)). Relatively higher IC_50_ (8.91*μ*M) of diethyldithiocarbamate was seen for CYP2E1 when chlorzoxazone was used as a probe. Phillygenin inhibited CYP2E1 activity with *K*_i_ of 2.40 *μ*M, and the Lineweaver-Burk plot (Figure 2(b)) was consistent with a competitive inhibition profile.

### 3.3. Hepatoprotective Effects of OFE/ Phillygenin on CCl4-Induced Mice Liver Injury

The effects of phillygenin/OFE on liver antioxidant status are presented in Table 1. The serum levels of hepatic enzymes AST and ALT, used as biochemical markers for evaluation of early hepatic injury, were significantly elevated (p < 0.01) in the CCl4-treated mice. Pretreatment with phillygenin or OFE significantly prevented the elevation of these marker enzymes (p < 0.05), compared to the negative control model group animals. A significant increase in MDA level (p < 0.01), an indicator of lipid peroxidation, was found in the livers of CCl4-intoxicated mice relative to normal mice. Pretreatment with different doses of phillygenin /OFE reversed this biochemical parameter significantly towards normal level (p < 0.05). The activities of antioxidant enzymes SOD in liver homogenates were significantly decreased (p < 0.01) in liver injury model groups when compared to normal controls. The results show that phillygenin/OFE exerts a beneficial effect on antioxidant enzymes (*p* < 0.01). The activities of AST, SOD, and MDA after treatment with the lowest dosage of phillygenin/OFE showed almost the same levels after treatment with bifendate, the potent hepatoprotective drug used as positive control [16–18].

**Figure 3:**
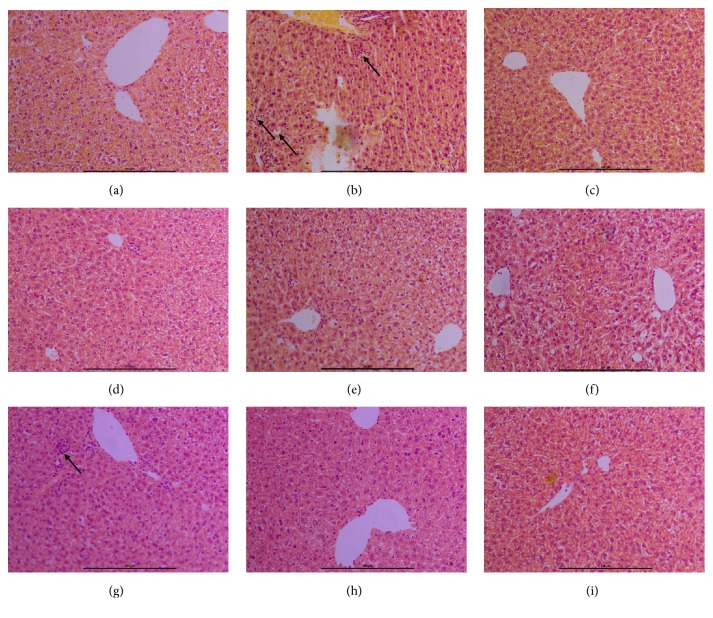
Effect of phillygenin/OFE on liver histopathology of CCl_4_-intoxicated mice. (a) Normal group liver section; (b) liver section of CCl_4_-intoxicated group; (c) liver section of bifendate + CCl_4_ treated group; (d) liver section of phillygenin (6 mg/kg) + CCl_4_-treated group; (e) liver section of phillygenin (12 mg/kg) + CCl_4_-treated group; (f) liver section of phillygenin (24 mg/kg) + CCl_4_-treated group. (g) Liver section of OFE (150 mg/kg) + CCl_4_-treated group; (h) liver section of OFE (300 mg/kg) + CCl_4_-treated group; (i) liver section of OFE (600 mg/kg) + CCl_4_-treated group. Original magnification 200× (a, b, c, d, e, and f).

The histological examination showed no pathological abnormalities in the liver of normal control animals (Figure 3(a)). However, histopathological analysis of the liver sections of CCl_4_-treated animals showed a moderate degree of centrilobular necrosis, hepatocyte ballooning, and infiltration of inflammatory cells into the portal tract and sinusoid in the necrotic lesion (Figure 3(b)). Pretreatment with phillygenin/OFE reversed the hepatic lesions produced by CCl_4_ as it is evident from the absence of cellular necrosis and inflammatory infiltrates in the liver section of treated mice (Figures 3(d)–3(i)), which were almost comparable to those of the normal control and bifendate (Figure 3(c)) treated groups.

### 3.4. Pharmacokinetics of Phillygenin

**Figure 4:**
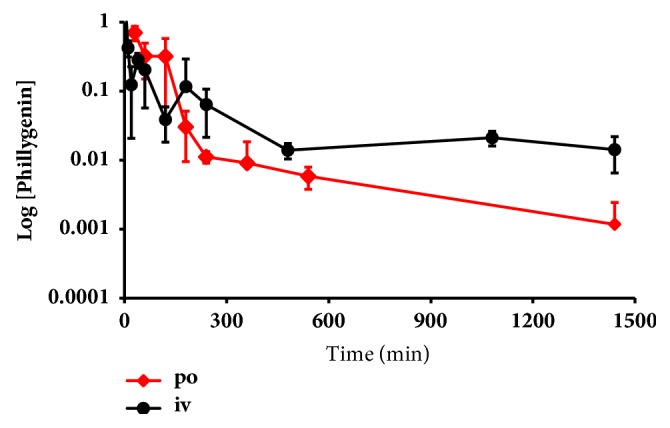
Plasma concentration-time curves after single oral dose administration of phillygenin (24 mg/kg) and single* iv* dose administration of phillygenin (12 mg/kg). Concentrations are given in *μ*g/mL for plasma.

The method developed (Supplementary Material available here) was successfully applied to determine the plasma concentrations of phillygenin in mice following oral (*po*, 24 mg / kg) or intravenous (*iv*, 12 mg/kg) administration. The plasma concentration-time profiles of phillygenin are presented in Figure 4, and their corresponding pharmacokinetic parameters are summarized in Table 2; the *AUC*_0-last_ is 72.5 min*∙μ*g/mL. The *AUC*_0-last_ of phillygenin after* iv* administration of 12 mg/kg is 63.2 min*∙μ*g/mL, pharmacokinetic parameters are presented in Table 2, and the plasma concentration versus time curves are shown in Figure 4. After oral administration, phillygenin was quickly absorbed, reaching maximum levels at 30 min. The phillygenin was slowly distributed to tissues, with a half-life of 150 min. The elimination a half-live was 240 min. Oral bioavailability of phillygenin in mouse is 56.4%.

### 3.5. Metabolism of Phillygenin in Mice

**Figure 5:**
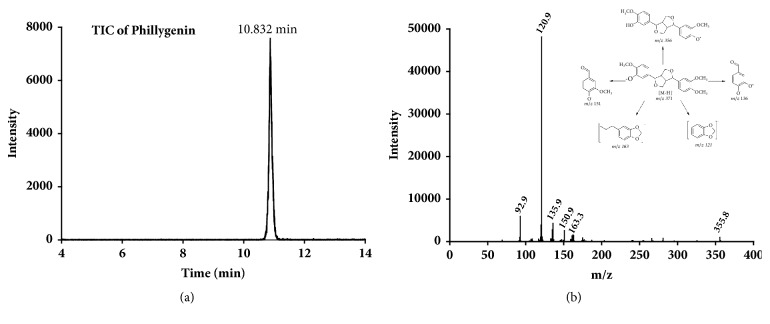
UPLC-MS/MS spectra of phillygenin. (a) Total ion chromatogram of phillygenin; (b) UPLC-MS/MS product ion spectrum of phillygenin.

The LC-MS and LC-MS/MS analyses of phillygenin were performed in electrospray negative ion mode. The full-scan mass spectrum of phillygenin gave protonated molecular ion [M - H]^−^ at m/z 371. The MS^2^ spectrum of the molecular ion contains five main product ions at m/z 356, 163, 151, 136, and 121. The proposed fragmentation of phillygenin was shown in Figure 5. The product ions and the corresponding neutral fragment loss were the characteristic structural information of phillygenin and were the sound basis to identify metabolites of phillygenin. Possible metabolite structures were considered based on the structure of phillygenin and known common metabolic pathways. Phillygenin and its two metabolites with their protonated molecular ions [M -H]^−^ at m/z 371, 387, and 357 were detected in mouse urine sample.

**Figure 6:**
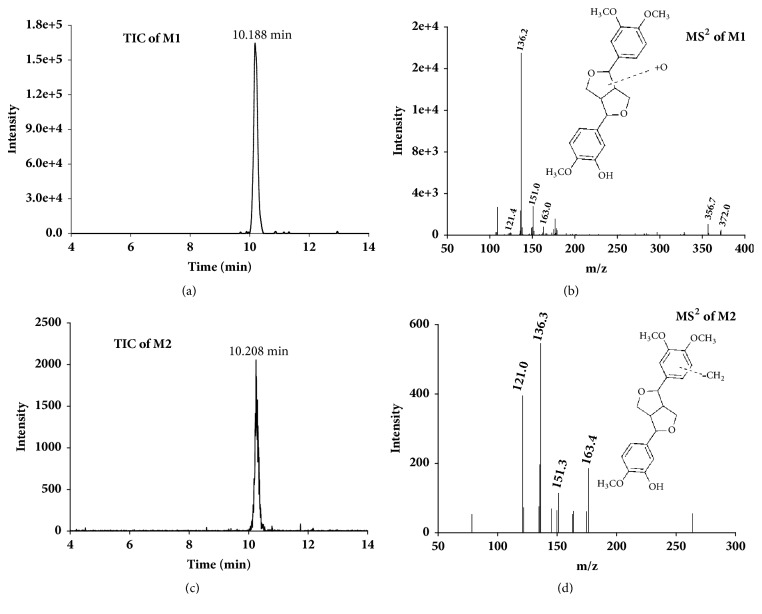
UPLC-MS/MS spectra of the metabolites of phillygenin. (a) Total ion chromatogram of M1; (b) UPLC-MS/MS product ion spectrum of M1; (c) total ion chromatogram of M2; (d) UPLC-MS/MS product ion spectrum of M2.

The molecular weight and MS^2^ fragmentation characteristic of each metabolite were compared with those of phillygenin for the more precise structural elucidation of the metabolites. Among them, the retention time and the MS^2^ of the molecular ion at m/z 371 (M0) were the same as those of phillygenin. Therefore, M0 can be affirmed as the unchanged phillygenin. The structures of metabolites were interpreted as follows. The molecular ion of M1 (m/z 387) and its main MS^2^ product ions at m/z 372, 356, 163,151, and 136 (Figure 6(b)) were almost the same as those of phillygenin, which means M1 might be the hydroxylated metabolite of phillygenin. The product ion of M2 at m/z 357 can lead to a characteristic MS^2^ product ion at m/z 163, 151, 136, and 121 (Figure 6(d)); compared with the fragment of phillygenin, M2 may be the demethylation metabolite of phillygenin.

## 4. Discussion

Hepatotoxicity induced by CCl_4_ is the most commonly used model system for the screening of hepatoprotective activity of plant extracts and drugs [10, 19].* Osmanthus fragrans*' petroleum ether extract showed a great hepatoprotective effect on CCl_4_-induced hepatic injury mice, and phillygenin is the main ingredient in* Osmanthus fragrans*' petroleum ether extract. The purpose of this study is to determine the hepatoprotective effect of phillygenin/OFE on the CCl_4_-induced liver injury, and the pharmacokinetics and metabolism of phillygenin were also performed.

ABTS and DPPH assays are commonly used to evaluate antioxidant activities of natural compounds in foods or biological systems. Ascorbic acid, used as a positive control, is a naturally occurring organic compound which can block some of the damage caused by free radicals [20, 21]. Antioxidant active assays show that phillygenin can bleach the DPPH and ABTS radical immediately and possess better effective ABTS radical-scavenging activity than ascorbic acid, which suggests that phillygenin could be classified as dynamic antioxidants.

CCl_4_ can be metabolized by CYP2E1 and produce free radical such as CCl_3_· and CCl_3_OO· which can bind to macromolecules and initiate the chain reaction of lipid peroxidation to cause liver cell damage [22]. Chemicals that reduce CYP2E1 activity may alleviate the toxicity caused by CCl_4_, and it was also expected that a radical scavenger may ameliorate CCl_4_ induced liver toxicity [22, 23]. A report indicates that CYP2E1 inhibitor but not antioxidants can totally or partially prevent CCl_4_ induced hepatic toxicity at an early time, suggesting that reducing CYP2E1 activity could be a better strategy than antioxidant to prevent CCl_4_-induced early hepatotoxicity [24]. Present study shows that phillygenin can inhibit the activity of CYP2E1 with *K*_i_ of 2.40 *μ*M. The results suggest that inhibition of the activity of CYP2E1 might be a mechanism of phillygenin that plays the hepatoprotective role in CCl_4_-induced early liver injury in mice.

The blood serum and liver enzymes (such as ALT and AST) were used as indicators of hepatic damage. The damaged liver cells can spill ALT and AST into blood, raising these two enzymes levels in blood and signaling the liver damage. Present study shows that CCl_4_-induced liver toxicity could lead to the increased activities of ALT and AST in serum that could be attributed to the liver damage resulting in the release of functional enzymes from hepatocytes. However, the increased levels of ALT and AST in serum were significantly decreased by pretreatment with phillygenin/OFE, implying that phillygenin/OFE may protect the hepatocytes against toxicity induced by CCl_4_. As the marker of lipid peroxidation, MDA levels in phillygenin/OFE group are almost the same as the normal control group, which means phillygenin/OFE can block the lipid peroxidation. Based on the current results, OFE appears to decrease MDA levels more than phillygenin, indicating other antioxidants in OFE. There is a report that there are five phenolic compounds in* O. fragrans*, which may contribute to decreasing MDA levels [3]. SOD is an intracellular antioxidant enzyme that protects the cell against oxidative stress. Phillygenin/OFE significantly decreases the activity of SOD in CCl_4_-induced mice liver and the levels are almost the same as the normal control group. Histopathological showed severe liver damage in CCl_4_ group mice such as centrilobular necrosis, hepatocyte ballooning, and infiltration of inflammatory cells into the portal tract and sinusoid in the necrotic lesion. Pretreatment with phillygenin/OFE can reverse the hepatic lesions produced by CCl_4_.

Although phillygenin has many pharmacological activities, there is only one report about the pharmacokinetics study of phillygenin in rat [25], which shows quick elimination after* iv *administration in the rat. There is limited information about the absorption and the first pass effect with which a drug is absorbed after oral administration, its bioavailability should be assessed. Pharmacokinetics study shows that phillygenin can be absorbed quickly with the absorption half-life of 54.8 min and efficiently with the bioavailability of approximately 56.4%. After absorption, phillygenin distributed quickly with the distribution half-life of 150 min and widely with the Vd of 300 mL/kg in mice. Two metabolites, hydroxylated and demethylation, were identified in rat urine sample after oral administration of phillygenin.

## 5. Conclusions

*Osmanthus fragrans* is a plant that has been used in traditional Chinese medicine to treat menopathies. The extract of* Osmanthus fragrans* flowers showed neuroprotective, free-radical scavenging, antioxidative effects* in vitro*. Phillygenin, one of the major ingredients in* Osmanthus fragrans*, shows great hepatoprotective effects on CCl_4_-induced liver injury in mice, antioxidant activity, and inhibition on CYP2E1* in vitro*. Meanwhile, as an oral administrated compound, phillygenin owns good bioavailability. These results suggested that phillygenin could be explored as new and potential natural antioxidants and hepatoprotective agents for use in functional foods.

## Figures and Tables

**Table 1:** Effect of phillygenin/OFE on AST, ALT, SOD, and MDA activities in blood serum of normal and experimental mice (n = 6).

	**ALT** **(nmol/(s L))**	**AST** **(nmol/(s L))**	**SOD** **(U/mg protein)**	**MDA** **(nmol/mg protein)**
**normal control**	42.0 ± 7.26	183 ± 16.8	52.8 ± 4.90	1.75 ± 0.482
**CCl**_**4 **_ **control**	271 ± 92.0^*∗∗*^	278 ± 35.6^*∗∗*^	39.2 ± 5.17^*∗∗*^	2.54 ± 0.251^*∗∗*^
**positive control**	70.0 ± 18.3^##^	186 ± 10.4^##^	47.2 ± 2.95^##^	1.81 ± 0.270^##^
**150 mg/kg OFE**	111 ± 43.1^##^	175 ± 15.5^##^	46.5 ± 4.25^#^	1.82 ± 0.541^#^
**300 mg/kg OFE**	109 ± 23.2^##^	171 ± 30.4^##^	45.9 ± 2.65^#^	1.79 ± 0.250^##^
**600 mg/kg OFE**	99.0 ± 25.7^##^	178 ± 24.9^##^	46.5 ± 3.63^#^	1.76 ± 0.430^##^
**6 mg/kg phillygenin**	111 ± 30.5^##^	190 ± 29.2^##^	46.4 ± 4.27^#^	1.85 ± 0.820
**12mg/kg phillygenin**	112 ± 23.1^##^	196 ± 28.4^##^	46.9 ± 2.74^##^	1.91 ± 0.224^##^
**24 mg/kg phillygenin**	110 ± 16.4^##^	192 ± 20.8^##^	49.5 ± 6.89^#^	1.92 ± 0.413^#^

*∗*  *p* < 0.05, *∗∗*  *p* < 0.01 versus normal control group.

^#^  *p *< 0.05, ^##^  *p* < 0.01, versus CCl_4_ control group.

**Table 2:** Pharmacokinetic parameters of phillygenin after single oral (24 mg/kg) or *iv* (12 mg/kg) to mice.

**Parameter**	***po* (24 mg/kg)**	***iv* (12 mg/kg)**
**A** **U** **C**_0-last_ **(** *μ*g·min/mL**)**	71.3	63.2
**A** **U** **C**_0-**∞**_ **(** *μ*g·min/mL**)**	72.5	76.2
**Vd (mL/kg)**	^*c*^ NC	300
**CL (mL/min/kg)**	345	156
**C**_0_ ** (**μ**g/mL)**	^*b*^NQ	3.14
**C**_**max**_ **(**μ**g/mL)**	0.7	- -
**T**_**max**_ ** (min)**	30	- -
**t**_(1/2)**α**_ ** (min)**	^*c*^ NC	3.78
**t**_(1/2)**β**_ ** (min)**	^*c*^NC	630
**A Half-life (min)**	54.8	- -
**D Half–life (min)**	150	^*a*^ NC
**E Half-life (min)**	240	^*a*^NC
**F (**%**)**	56.4	- -

^*a*^NC = not calculated; low levels did not allow calculation of terminal half-life and *AUC*_last-*∞*_.

## Data Availability

The data used to support the findings of this study are available from the corresponding author upon request.

## Abbreviations

CCl4: Carbon tetrachloride
OFE: Osmanthus fragrans flower extract
CYP2E1: Cytochrome P450 2E1
EDTA: Ethylenediamine tetracetic acid
DPPH: Diphenyl-2-picrylhydrazyl
NADPH: *β*-Nicotinamide adenine dinucleotide 2′-phosphate reduced tetrasodium salt hydrate
UPLC: Ultra-performance liquid chromatography
AST: Aspartate aminotransferase
alanine ALT: Aminotransferase
SOD: Superoxide dismutase
MDA: Malondialdehyde
QC: Quality control
AUC: The area under the mean concentration-time curve
Vd: Volume distribution
Cl: Clearance
C_0_: Initial concentration.

## Supplementary Materials

Supplementary Materials Figure S1 shows the specificity of developed method for phillygenin quantification. Tables S1 gives the precision, accuracy, extraction recovery, and matrix effects of phillygenin in spiked mouse plasma, respectively. Table S2 reports the stability of phillygenin in mouse plasma under various conditions.
