# Supplementary material for: Evaluation of the Pharmacokinetics and Hepatoprotective Effects of Phillygenin in Mouse
Source: Biomed Res Int. 2018 Aug 23;2018:7964318. doi: 10.1155/2018/7964318 (PMC6126057; doi:10.1155/2018/7964318)
Supplement: Supplementary Materials — Figure S1 shows the specificity of developed method for phillygenin quantification. Tables S1 gives the precision, accuracy, extraction recovery, and matrix effects of phillygenin in spiked mouse plasma, respectively. Table S2 reports the stability of phillygenin in mouse plasma under various conditions. [file 7964318.f1.pdf]

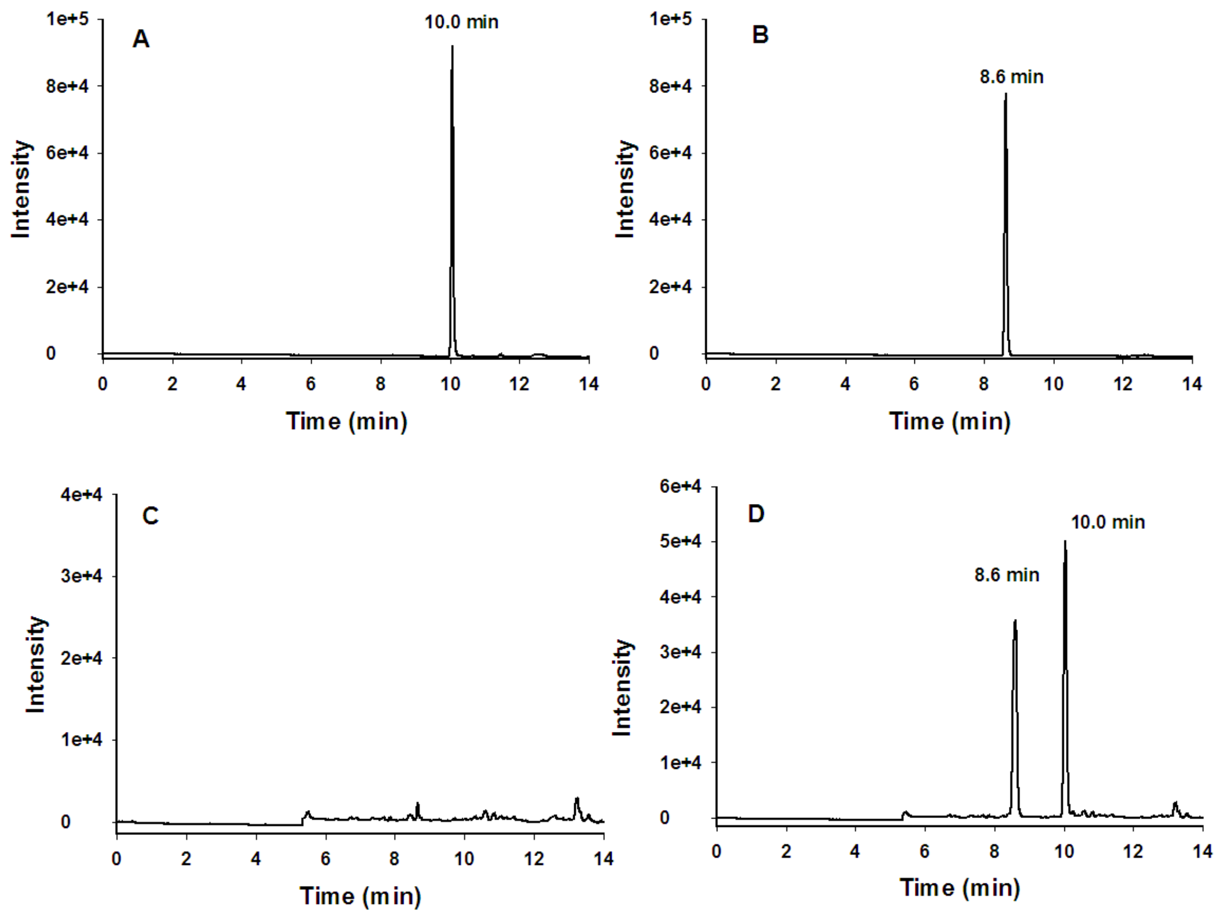

Figure. S1. UPLC-MS/MS of (A) standard phillygenin (10μg/mL), (B) standard phenacitin (50 μg/mL), (C) blank mouse plasma, and (D) blank mouse plasma spiked with standard phillygenin (10 μg/mL) and internal standard (Phillygenin, retention time = 10.0 min; internal standard, retention time = 8.6 min).

Table S1. Precision, accuracy, extraction recovery and matrix effects for the determination of phillygenin in mouse plasma (n = 6).

**Concentration**	**Intra-day**		**Inter-day**		**Extraction recovery**		**Matrix effect**	
	RSD (%)	Accuracy (%)	RSD (%)	Accuracy (%)	Mean ±SD (%)	RSD (%)	Mean ±SD (%)	RSD (%)
**0.01**	6.41	103.3	12.9	92.6	101 ± 3.27	6.94	89.6 ± 4.02	5.42
**1**	6.86	103.7	5.42	106	103 ± 2.58	4.86	96.2 ± 3.64	6.98
**10**	3.53	102.3	2.36	101	101± 2.03	2.97	102 ± 3.95	7.52

Table S2. Stability of phillygenin in mouse plasma under various conditions (n = 6)

**Stability conditions**	**Concentration (µg/mL)**	**Calculated concentration**		
		Mean ± SD (µg/mL)	RSD (%)	Accuracy (%)
**Short-term (RT, 4h)**	0.01	0.0090 ± 0.0021	10.30	90.10
	1	0.98 ± 0.0058	6.56	98.01
	10	9.65 ± 0.72	3.26	96.51
**Long-term**	0.01	0.0092 ± 0.0026	7.86	92.15
	1	1.03 ± 0.704	8.41	103.12
	10	10.01 ± 0.63	5.33	100.14
**Freeze-thaw cycles (from -80 ℃ to RT)**	0.01	0.0954 ± 0.0007	9.64	95.44
	1	1.02 ± 0.050	6.84	102.31
	10	10.12 ± 0.231	2.97	101.20
